# Deep Learning Based Evaluation of Skeletal Maturation: A Comparative Analysis of Five Hand‐Wrist Methods

**DOI:** 10.1111/ocr.70008

**Published:** 2025-07-24

**Authors:** Serhat Tentaş, Samet Özden

**Affiliations:** ^1^ Department of Orthodontics Faculty of Dentistry, İnönü University Battalgazi, Malatya Türkiye

**Keywords:** artificial intelligence, bone age, deep learning, skeletal maturation

## Abstract

**Objective:**

The study aims to evaluate the effectiveness of deep learning algorithms in skeletal age estimation by comparing the diagnostic reliability of five different hand‐wrist maturation (HWM) assessment methods.

**Materials and Methods:**

A total of 6572 hand‐wrist radiographs from orthodontic patients aged 8–16 years were retrospectively analysed. Radiographs were categorised into five groups based on HWM classification methods: (I) Björk's nine‐stage, (II) Hägg and Taranger's five‐stage, (III) Chapman's four‐stage, (IV) three‐stage hook of hamate ossification based and (V) simplified three‐stage Björk's classification based. YOLOv8x‐based deep learning models were trained separately for each group. The dataset was split into training, validation and test subsets. Performance was evaluated using accuracy, precision, recall, F1 score and AUC metrics.

**Results:**

The YOLOv8x‐cls model demonstrated high classification performance across all five groups. Group IV and Group II achieved the highest accuracy and F1 scores, with average F1 values of 0.99 and 0.96, respectively. Group III and Group V also showed strong performance (F1 = 0.93 and 0.92). In Group I, slightly lower classification performance was observed in the S‐H2 and MP3‐Cap stages (F1 = 0.72–0.74), which correspond to the pubertal growth peak, while early and late skeletal maturation stages were classified with high accuracy and F1 scores. ROC curve analysis further supported these findings, with AUC values for MP3‐Cap and S‐H2 recorded as 0.70 and 0.75, respectively, whereas higher AUC values were achieved in most other stages across all groups.

**Conclusion:**

Deep learning models proved effective in evaluating skeletal maturation across five different HWM methods. Particularly high performance was observed in anatomically distinct regions such as the MP3, adductor sesamoid and hamate bone, which can be reliably identified by general dentists, enabling earlier referrals and timely orthodontic interventions.

## 1 Introduction

The assessment of skeletal maturation is essential in orthodontics and paediatric dentistry; it determines the optimal timing for growth‐modification treatments [1]. Particularly in patients with maxillomandibular skeletal discrepancies, treatment success depends on precise evaluation, since jaw growth redirection is only effective at specific developmental stages [2]. Chronological age alone is unreliable for determining growth potential due to individual variations; therefore, skeletal development, dental age, weight and sexual maturation must be considered [3, 4]. Radiographic methods, including bone age assessment and skeletal maturation staging, remain fundamental for estimating growth timing and velocity. Recently, artificial intelligence (AI) has emerged as a promising tool, offering enhanced accuracy and consistency in skeletal maturation evaluation. A comprehensive understanding of a patient's growth trajectory enables more effective and predictable orthodontic interventions, and integrating advanced technologies with conventional diagnostics could further refine treatment strategies, ensuring optimal outcomes.

Throughout history, various methods have been utilised to assess skeletal maturation stages, each evolving to enhance diagnostic accuracy and clinical applicability. Among these, cervical vertebral maturation (CVM) [5, 6] and hand‐wrist maturation (HWM) classifications are the most widely recognised techniques. Although the cervical CVM method is a widely used approach HWR's offer a more detailed evaluation by incorporating multiple ossification centres and epiphyseal fusion patterns. The complex structure of the hand and wrist bones allows for simultaneous assessment of numerous bones, providing a more precise estimation of skeletal maturation. This comprehensive approach minimises interpretation errors and enhances accuracy, particularly when integrated with AI for automated analysis.

Various methods have been developed for skeletal age estimation using HWR's. Björk's [7] 9‐stage classification examines different regions of the hand and wrist, while Fishman's [8] 11‐stage system utilises Skeletal Maturation Indicators (SMI) for a structured assessment. More compact approaches include Hagg and Taranger's [9] five‐stage system, which evaluates the epiphysis–diaphysis relationship in the third finger, and Chapman's [10] classification, based solely on the adductor sesamoid (AS) bone.

The integration of AI into skeletal maturation assessment has significantly enhanced the accuracy and efficiency of age estimation by minimising observer variability and enabling automated analysis of radiographic images. This advancement is particularly valuable in clinical settings where general dentists, who are often the first point of contact for growing patients, may lack the expertise to accurately interpret HWR's. AI‐assisted systems provide a reliable and accessible tool for evaluating skeletal maturity, allowing general practitioners to make informed referrals to orthodontists. As a result, patients can be directed to specialised care at the optimal time, ensuring that orthopaedic treatments are initiated before the critical growth phases conclude, ultimately improving treatment outcomes.

Although numerous studies have utilised AI‐driven machine learning [4, 11] and deep learning [12, 13, 14] algorithms for HWM assessment, to the best of our knowledge, no study has simultaneously compared the accuracy of ‘five different HWM techniques' using such a large dataset of HWR's. Addressing this gap, the present study aims to evaluate the performance of deep learning algorithms in skeletal age estimation while assessing the reliability of multiple HWM methods.

## 2 Material and Methods

### 2.1 Ethical Approval and Patient Selection

This retrospective study received ethical approval from Inonu University Non‐Interventional Clinical Research Ethics Committee (Numbered: 2023/5340, Dated: 26.12.2023), confirming adherence to ethical guidelines. The study included a total of 14 210 HWR's obtained from patients aged 8–16 years who underwent orthodontic treatment at the Department of Orthodontics, Faculty of Dentistry, Inonu University between 2010 and 2024.

To ensure data integrity and standardisation, radiographs were excluded if they met any of the following criteria: (1) individuals of non‐Turkish nationality, (2) radiographs obtained from the right hand, (3) history of hand‐wrist trauma, (4) systemic metabolic disorders affecting bone development, (5) prolonged infectious diseases, (6) syndromic conditions impacting growth and development, (7) anatomical variations in the hand‐wrist region and (8) poor image quality or presence of artefacts.

Following the application of these criteria, 7658 radiographs were excluded, resulting in a final dataset of 6572 high‐quality left‐hand wrist radiographs for further analysis.

### 2.2 Study Groups and Labelling of Hand‐Wrist Radiographs

All 6572 HWR's were obtained using the same radiographic device (Planmeca OY, Helsinki, Finland) to ensure standardisation. Device settings were adjusted based on patient age and weight, averaging 60 kV and 2 mA. Radiographs were taken with proper hand positioning, ensuring visible distal radius, ulna and fingertips. All images were uploaded to the CranioCatch software (Eskişehir, Türkiye), classified by skeletal maturation method and labelled for regions of interest (ROIs). To reduce interobserver variability, all classification and labelling were performed by a single examiner (S.Ö).

The ‘five study groups’ established in this research and the corresponding analytical procedures applied to each are outlined as follows:

#### 2.2.1 Group I

Classified according to Björk's [7] nine‐stage maturation method which evaluates all regions of the HWR (Table 1). In this group, no manual labelling was performed, as the deep learning model was designed to simultaneously analyse multiple regions of interest within the HWR's.

**TABLE 1:** Skeletal maturation stages based on Björk's, Hägg's, Tranger's and Chapman's HWM classification.

No	Stage	Björk's nine‐stage classification description
1	PP2	The epiphyseal–diaphyseal equality is observed in the proximal phalanx of the 2nd finger.
2	MP3	The epiphyseal–diaphyseal equality is observed in the medial phalanx of the 3rd finger.
3	Pisi/H1/R	The Pisiform bone begins to calcify, and the hook of the hamate bone becomes visible. Epiphyseal–diaphyseal equality is observed in the radius. The pubertal growth spurt initiates during this stage.
4	S‐H2	The adductor sesamoid bone becomes visible on the radiograph, and the hook of the hamate bone is clearly distinguishable. The pubertal growth spurt approaches its peak.
5	MP3‐Cap	The epiphysis of the medial phalanx of the 3rd finger has begun to cap over the diaphysis, indicating the peak of the pubertal growth spurt.
6	DP3U	The epiphysis and diaphysis of the distal phalanx of the 3rd finger have fused. The pubertal growth spurt has begun to slow down.
7	PP3U	The epiphysis and diaphysis of the proximal phalanx of the 3rd finger have fused.
8	MP3U	The epiphysis and diaphysis of the medial phalanx of the 3rd finger have fused.
9	RU	The epiphysis and diaphysis of the radius have fused, indicating the complete cessation of skeletal growth potential.

No	Stage	Hägg and Taranger's five‐stage MP3 classification description
1	MP3‐F	The pubertal growth spurt has not yet begun, and epiphyseal–diaphyseal equality is observed.
2	MP3‐FG	The growth acceleration phase, where the epiphysis and diaphysis remain equal in size, and protuberances appear on the lateral and medial regions of the epiphysis.
3	MP3‐G	The peak of the pubertal growth spurt. The epiphysis thickens, develops prominent edges and begins capping over the diaphysis.
4	MP3‐H	The growth rate starts to decrease, and fusion between the epiphysis and diaphysis begins.
5	MP3‐I	The pubertal growth spurt is complete, with full fusion of the epiphysis and diaphysis.

No	Stage	Chapman's four stage AS bone classification description
1	AS‐0	No calcification is observed in the region of the adductor sesamoid bone.
2	AS‐1	A calcification of approximately 1 mm in diameter appears in the adductor sesamoid bone and remains visible for about 3 months, signalling the onset of the pubertal growth spurt.
3	AS‐2	Calcification progresses, but the borders remain indistinct. This stage lasts approximately 6 months.
4	AS‐3	The adductor sesamoid bone reaches its final size and ultimate calcification stage, with clearly defined borders. The borders of the adductor sesamoid bone become clearly defined.

#### 2.2.2 Group II

**FIGURE 1:**
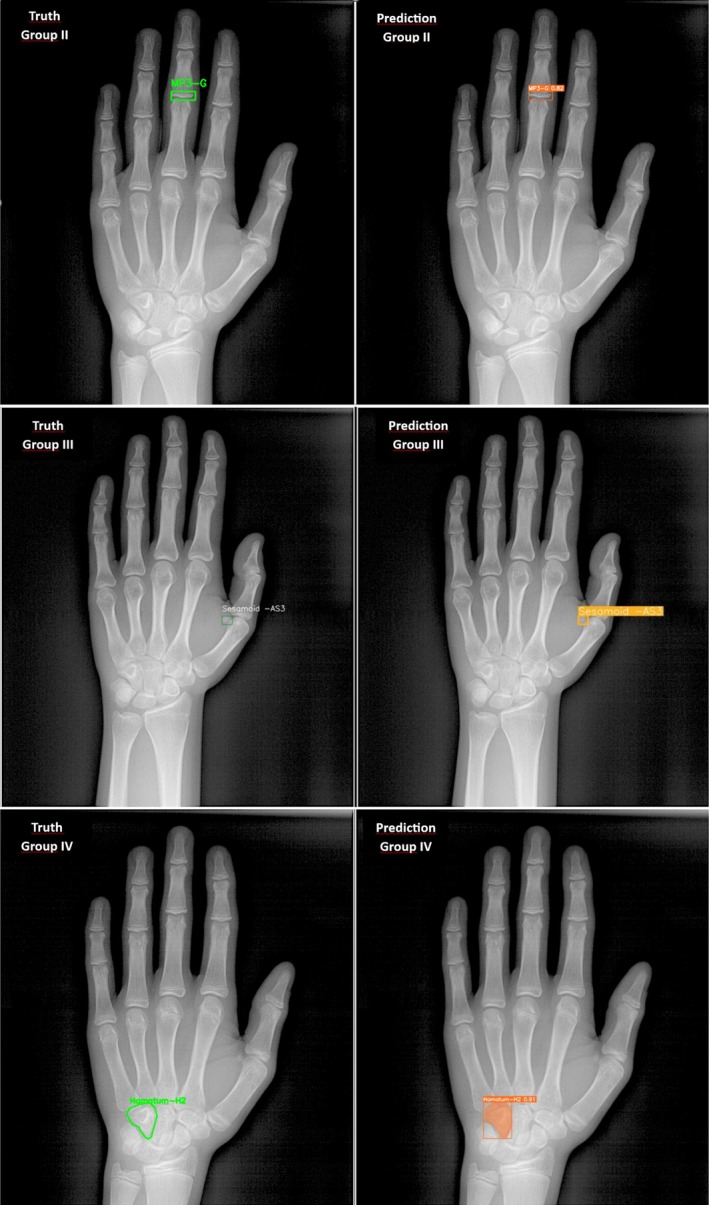
Representative examples of truth and predicted skeletal maturation stage labelling for Group II, Group III and Group IV. Each image illustrates the ground‐truth labels (actual maturation stage) alongside the AI‐predicted labels for the corresponding hand‐wrist radiographs.

Categorised using Hägg and Taranger's [9] five‐stage maturation method (Table 1) which focuses on the medial phalanx of the third finger. In this group, the MP3 region was labelled using a ‘rectangular detection module’ to enhance the deep learning model's focus on the relevant area for skeletal maturation assessment. (Figure 1).

#### 2.2.3 Group III

Categorised based on Chapman's [10] four‐stage maturation method, which assesses the AS bone (Table 1). In this group, labelling using a ‘rectangular detection module’ was applied to the AS bone to guide the deep learning model's focus on the relevant region for skeletal maturation assessment. (Figure 1).

#### 2.2.4 Group IV

A modified version of Björk's [7] classification, incorporating the H1 and H2 stages (Table 1), with an additional H0 stage representing the absence of calcification in the hook of the hamate bone on radiographs. In this group, labelling was applied to the hamate bone, encompassing its boundaries in a ‘polygonal format’, to guide the deep learning model's focus on the relevant region for skeletal maturation assessment. (Figure 1).

#### 2.2.5 Group V

Derived from Björk's [7] nine‐stage maturation system, where radiographs were reclassified into three subgroups: pre‐peak period, peak period and post‐peak period (Table 1). In this group, no manual labelling was performed, as the deep learning model was designed to simultaneously analyse multiple regions of interest within the HWR's.

### 2.3 Training of the Deep Learning Model and Dataset Preparation

**FIGURE 2:**
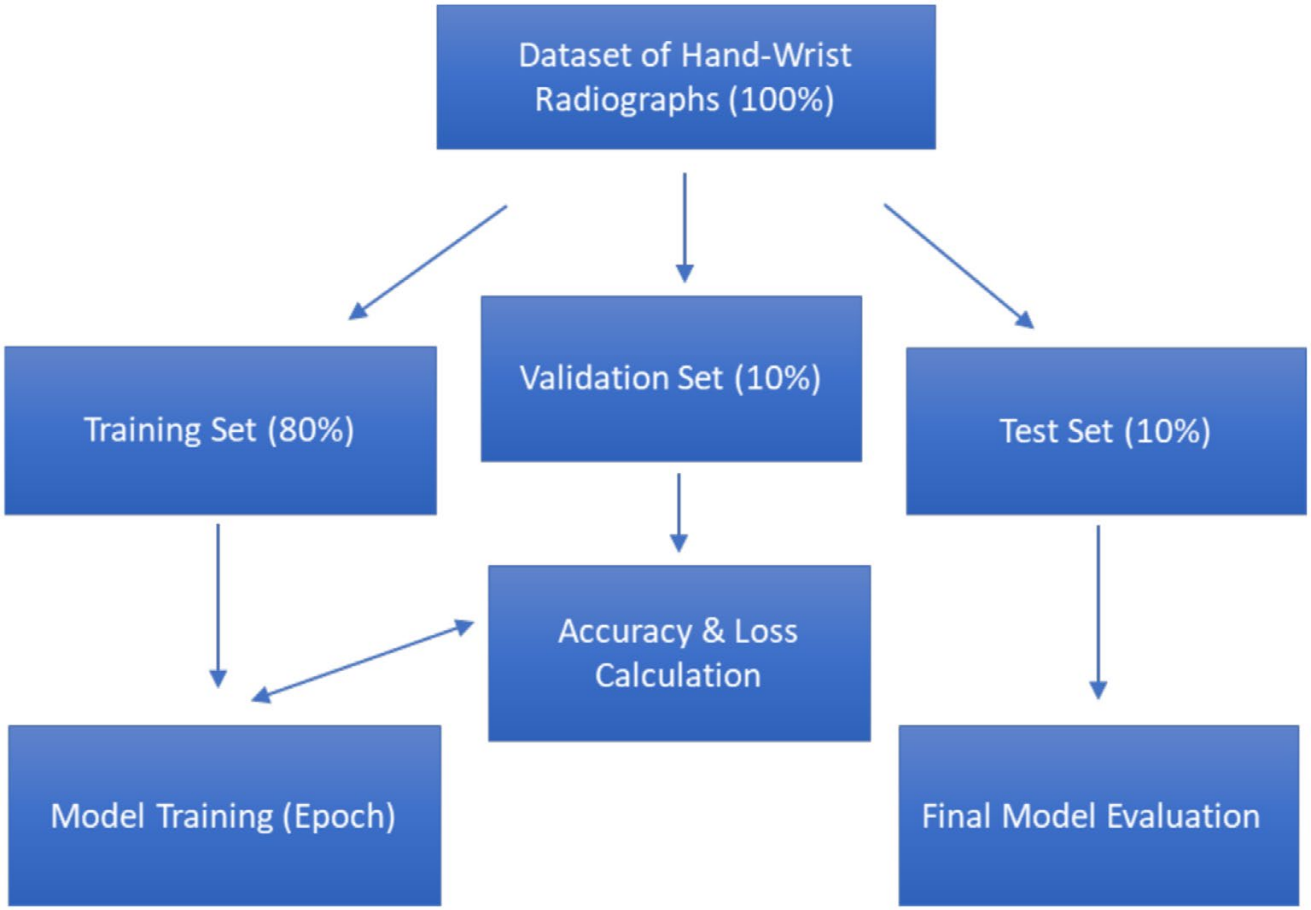
Schematic representation of the dataset division and model training workflow. A bidirectional connection between model training and accuracy & loss calculation illustrates the iterative feedback mechanism, where validation results influence model adjustments. After training, the final model evaluation was conducted using the test set to assess generalisation performance.

Model training was conducted on a system equipped with NVIDIA Tesla V100 GPU and 16 GB of RAM. A learning rate of 0.01 was used with the SGD optimizer, and Xavier initialisation was applied for weight initialisation. Each dataset was randomly and independently split into training (80%), validation (10%) and test (10%) subsets (Figure 2).

A learning rate of 0.01 was used, and learning rate scheduling was implemented with a final learning rate multiplier (lrf) of 0.01. Default data augmentation techniques—hsv_h, hsv_s, hsv_v, translate, scale, fliplr, mosaic, erasing—were applied to increase the robustness of the classification task. Prior to model input, image pixel values were normalised from a range of 0–255 to 0–1. Cross‐Entropy Loss function was used as the objective function, as it effectively measures the divergence between predicted and true class distributions in multi‐class classification. Regularisation was performed using weight decay (L2 regularisation) with a coefficient of 0.0005, and batch normalisation was applied (epsilon = 1e‐3, momentum = 0.03) to stabilise and accelerate training.

YOLOv8x‐seg and YOLOv8x‐cls models, based on the YOLOv8 architecture and implemented in the PyTorch library, were employed as the deep learning framework.

**FIGURE 3:**
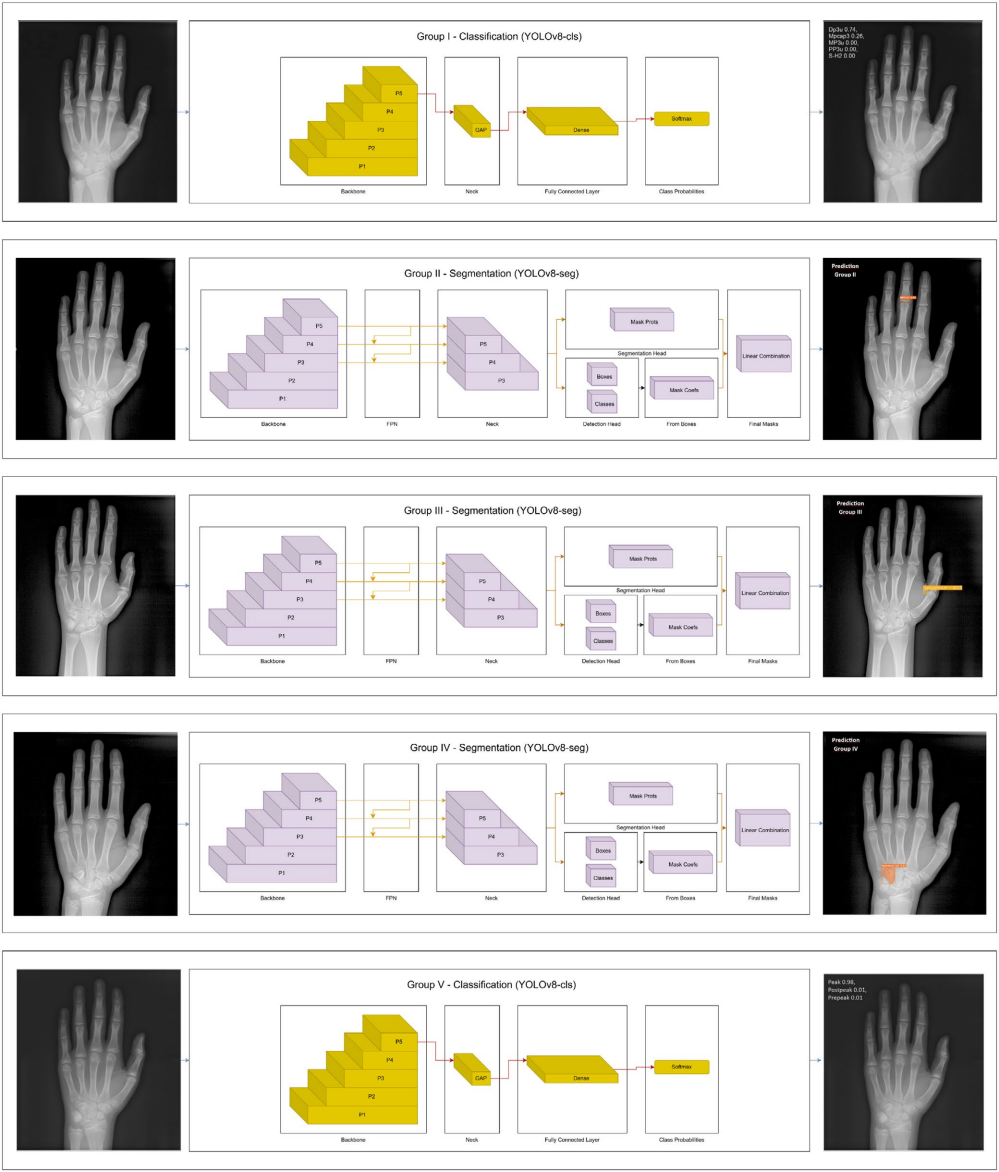
Schematic overview of the deep learning workflows applied in the five study groups. Groups I and V used a classification‐based approach (YOLOv8‐cls) analysing the entire radiograph, while Groups II, III and IV employed a segmentation‐based approach (YOLOv8‐seg) focusing on specific regions: MP3, adductor sesamoid and hamate bone, respectively.

‘Group I’ and ‘Group V’ utilised a classification‐based approach using the ‘YOLOv8x‐cls model’, where full hand‐wrist radiographs were input into the YOLOv8 backbone, followed by global average pooling (GAP), a fully connected dense layer, and a softmax classifier to generate class probabilities corresponding to skeletal maturation stages. No region‐of‐interest (ROI) segmentation was applied in these groups, as the algorithm was designed to focus on the entire hand‐wrist radiograph for the classification (Figure 3).

‘Groups II, III and IV’ employed a segmentation‐based strategy utilising the ‘YOLOv8x‐seg model’, in which hand‐wrist radiographs were processed through the YOLOv8 backbone, followed by a feature pyramid network (FPN), segmentation head, and classification layers with linear operations. In these groups, the model was directed to specific anatomical regions of interest: the MP3 region (Group II), the adductor sesamoid bone (Group III) and the hamate bone (Group IV) (Figure 3). The segmentation framework enabled region‐specific feature extraction based on these predefined skeletal landmarks prior to classification.

Figure 3 illustrates the overall deep learning workflow applied in each of the five study groups.

### 2.4 Performance Metrics for Evaluating the Deep Learning Model

To assess the performance of the deep learning models on the test dataset, a confusion matrix was utilised. The confusion matrix provides a comparative analysis between actual ground‐truth values and the model's predictions, offering insight into classification accuracy. The effectiveness of the deep learning model was evaluated using Accuracy, Precision, Recall and F1 Score, which were computed based on True Positives (TP), False Negatives (FN), False Positives (FP) and True Negatives (TN). The mathematical formulations of these metrics are as follows:

#### 2.4.1 Accuracy (Overall Correct Classification Rate)

The proportion of correctly predicted cases among the total number of cases, calculated as:

$$\text{Accuracy}=\left(\mathrm{TP}+\mathrm{TN}\right)/\left(\mathrm{TP}+\mathrm{TN}+\mathrm{FP}+\mathrm{FN}\right)$$

#### 2.4.2 Precision (Positive Predictive Value)

The ratio of correctly predicted positive cases to all predicted positive cases, computed as:

$$\text{Precision}=\mathrm{TP}/\left(\mathrm{TP}+\mathrm{FP}\right)$$

#### 2.4.3 Recall (Sensitivity)

The proportion of actual positive cases that were correctly identified by the model, given by:

$$\text{Recall}=\mathrm{TP}/\left(\mathrm{TP}+\mathrm{FN}\right)$$

#### 2.4.4 F1 Score

The harmonic mean of Precision and Recall, balancing false positives and false negatives, is calculated as:

$$\text{F1 Score}=\left(2\times \mathrm{TP}\right)/\left(2\times \mathrm{TP}+\mathrm{FP}+\mathrm{FN}\right)$$

Higher F1 scores indicate better classification performance, particularly in imbalanced datasets.

To further analyse the model's classification capability, the Receiver Operating Characteristic (ROC) curve was used. ROC is derived by plotting Recall (Sensitivity) against Precision to assess the trade‐off between true positive and false positive rates. This metric is particularly useful in cases where the dataset is imbalanced. The Area Under the Curve (AUC) of the ROC curve was also computed, ranging between 0 and 1, with higher values signifying better model performance in distinguishing between different classes. Alongside the confusion matrix, AUC values served as a key measure of the deep learning model's classification efficiency.

### 2.5 Measurement Error

For each dataset, skeletal maturation assessment was conducted by an orthodontist (S.Ö) with extensive clinical experience in orthodontics, who was blinded to patient information and chronological age. To evaluate intrarater reproducibility, measurements were repeated on 1314 randomly selected HWR's (20% of all samples). The same operator reassessed the radiographs one month after the initial evaluation.

## 3 Results

The final model's performance was evaluated using a test dataset of HWR's from 6572 patients (3112 males, 3460 females). The patients' ages at the time of radiograph acquisition ranged from 8 to 16 years, with a mean age of 12.54 ± 1.93 years.

The intraclass correlation coefficient (ICC) analysis demonstrated an almost perfect agreement between the first and second measurements in HWM stage assessment. The mean ICC values for each group were as follows: 0.934 for Group I (nine‐stage classification average), 0.947 for Group II (five‐stage classification average), 0.933 for Group III (four‐stage classification average), 0.967 for Group IV (three‐stage classification average) and 0.979 for Group V (three‐stage classification average).

**FIGURE 4:**
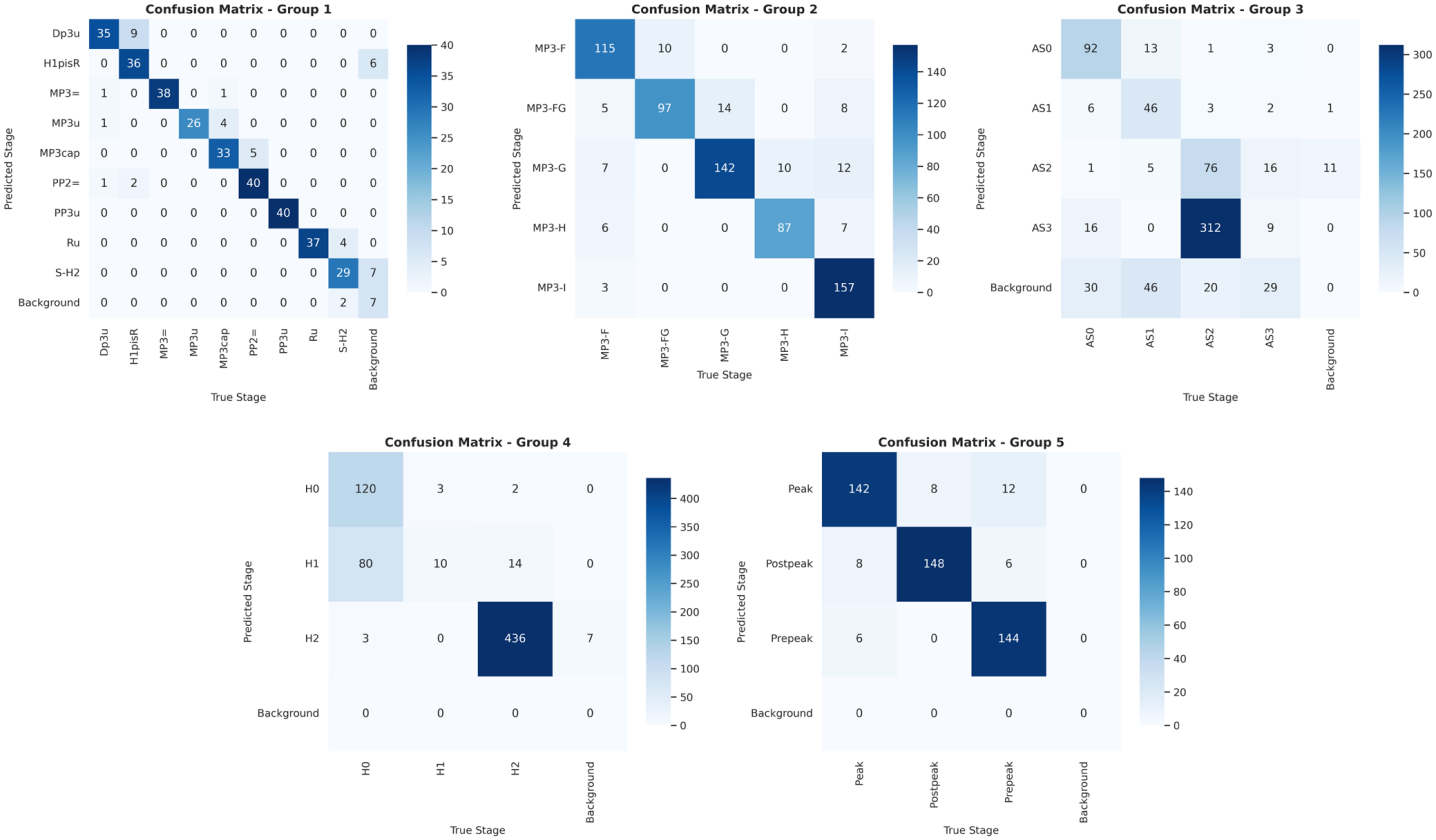
Combined confusion matrices for the five skeletal maturation assessment methods used in this study. The diagonal values represent correctly classified instances, while off‐diagonal values indicate misclassifications between different maturation stages.

Figure 4 presents representative confusion matrices for the four study groups, illustrating the AI model's performance across skeletal maturation stages. Diagonal elements indicate correctly classified instances (TP and TN), with higher concentrations reflecting stronger classification accuracy. Off‐diagonal values represent misclassifications (FP and FN), often occurring between adjacent stages. These patterns highlight stages that are more challenging to distinguish. In some cases, instances were misclassified as background, indicating the model's uncertainty in assigning a valid maturation stage.

Table 2 presents a comparative evaluation of the YOLOv8x‐cls model's performance across all groups, based on accuracy, precision, recall and F1 score.

**TABLE 2:** Performance metrics of the deep learning model for skeletal maturation classification across five study groups.

	No	Stages	Accuracy	Precision	Recall	F1 score	Deep learning model
Group I	1	PP2=	0.97	0.97	0.97	0.97	
	2	MP3=	0.97	0.95	0.97	0.95	
	3	Pisi/H1/R=	0.82	0.86	0.82	0.83	
	4	S‐H2	0.75	0.71	0.75	0.72	
	5	MP3‐Cap	0.70	0.80	0.70	0.74	
	6	DP3U	0.82	0.78	0.82	0.80	YOLO v8x‐cls
	7	PP3U	1.0	0.85	1.0	0.91	
	8	MP3U	0.75	0.93	0.75	0.83	
	9	RU	0.95	0.88	0.95	0.91	
	General		0.86	0.85	0.85	0.85	
Group II	1	MP3‐F	0.97	0.97	1	0.98	
	2	MP3‐FG	0.88	0.88	1	0.93	
	3	MP3‐G	0.90	0.90	1	0.95	YOLO v8x‐seg
	4	MP3‐H	0.86	0.86	1	0.92	
	5	MP3‐I	0.96	0.96	1	0.98	
	General		0.92	0.92	1	0.96	
Group III	1	AS‐0	0.86	0.96	0.89	0.92	
	2	AS‐1	0.73	0.77	0.93	0.84	
	3	AS‐2	0.85	0.87	0.97	0.92	YOLO v8x‐seg
	4	AS‐3	0.95	1	0.95	0.97	
	General		0.87	0.92	0.94	0.93	
Group IV	1	H‐0	0.99	0.99	1	0.99	
	2	H‐1	0.96	0.96	1	0.98	
	3	H‐2	0.98	0.98	1	0.99	YOLO v8x‐seg
	General		0.98	0.98	1	0.99	
Group V	1	Pre‐Peak	0.92	0.96	0.92	0.93	
	2	Peak	0.91	0.87	0.91	0.88	
	3	Post‐Peak	0.94	0.94	0.94	0.94	YOLO v8x‐cls
	General		0.92	0.92	0.92	0.92	

For Group I, PP2 = and MP3 = stages demonstrated high classification performance, each achieving 0.97 accuracy, with consistently strong precision, recall and F1 scores (PP2 = 0.97, MP3 = 0.95). Pisi/H1/*R* = and DP3U also exhibited high accuracy (0.82), with F1 scores of 0.83 and 0.80, respectively. The S‐H2 and MP3‐Cap stages showed relatively lower classification performance, with accuracies of 0.75 and 0.70 and corresponding F1 scores of 0.72 and 0.74, suggesting increased classification variability. PP3U achieved perfect classification performance, with 1.00 accuracy, 1.00 recall and an F1 score of 0.91, indicating the model's exceptional reliability in this category. MP3U and RU showed high classification performance, with 0.75 and 0.95 accuracy and F1 scores of 0.83 and 0.91, respectively. The overall model performance across all maturation stages in Group I resulted in 0.86 accuracy, 0.85 precision, 0.85 recall and 0.85 F1 score, highlighting the model's strong and reliable classification capability.

For Group II, MP3‐F and MP3‐I stages demonstrated high classification performance, each achieving 0.97 and 0.96 accuracy, with perfect recall (1.00) and F1 scores of 0.98. MP3‐FG and MP3‐G also exhibited high accuracy (0.88 and 0.90, respectively), with perfect recall and F1 scores of 0.93 and 0.95, indicating strong classification reliability. MP3‐H stage achieved a slightly lower accuracy of 0.86 but maintained perfect recall and an F1 score of 0.92, showing that despite minor classification variability. The overall model performance across all maturation stages in Group II resulted in 0.92 accuracy, 0.92 precision, 1.00 recall and 0.96 F1 score, highlighting the model's strong and highly reliable classification capability.

For Group III, AS‐3 stage demonstrated the highest classification performance, achieving 0.95 accuracy, perfect precision (1.00), 0.95 recall and an F1 score of 0.974, indicating highly reliable prediction. AS‐0 and AS‐2 also exhibited high classification accuracy (0.867 and 0.852, respectively), with corresponding F1 scores of 0.928 and 0.920, reflecting strong classification stability. The AS‐1 stage showed relatively lower accuracy (0.734), despite maintaining a high recall of 0.930, resulting in an F1 score of 0.846, suggesting greater classification variability. The overall model performance across all maturation stages in Group III resulted in 0.878 accuracy, 0.929 precision, 0.940 recall and 0.935 F1 score, highlighting strong classification capability with consistently high predictive accuracy.

For Group IV, the AI model exhibited exceptional classification performance across all stages, with H0 achieving near‐perfect accuracy (0.991), precision (0.991), perfect recall (1.00) and an F1 score of 0.995. Similarly, H1 and H2 demonstrated high accuracy (0.967 and 0.982, respectively), both maintaining perfect recall and achieving F1 scores of 0.983 and 0.990, respectively. The overall model performance in Group IV resulted in 0.981 accuracy, 0.981 precision, 1.00 recall and 0.990 F1 score, highlighting a near‐perfect classification capability in skeletal maturation prediction.

For Group V, the post‐pubertal stage demonstrated high classification performance, achieving 0.94 accuracy, 0.94 precision, 0.94 recall and an F1 score of 0.94, indicating strong predictive reliability. The pre‐pubertal stage also exhibited high accuracy (0.92) with an F1 score of 0.93, maintaining stable classification performance. The pubertal stage, while showing slightly lower precision (0.87), still achieved 0.91 accuracy, 0.91 recall and an F1 score of 0.88, reflecting consistent model performance. The overall model performance in Group V resulted in 0.92 accuracy, 0.92 precision, 0.92 recall and 0.92 F1 score, demonstrating strong and balanced classification capability.

Figure S1 displays the ROC curves for all study groups, illustrating the model's discriminative performance. The area under the curve (AUC), representing the overall ability of the model to distinguish between classes, approaches 1.0 for high sensitivity and specificity, while values near 0.5 indicate poor discrimination. Group IV stages H0 (AUC = 0.99), H1 (0.97) and H2 (0.98) showed near‐perfect classification. In Group II, MP3‐F (0.98) and MP3‐I (0.97) exhibited excellent diagnostic performance. Group III stages AS‐2 (0.97) and AS‐3 (0.95) also yielded excellent AUC values. Group I showed strong results for PP3U (1.00), PP2 = (0.98) and RU (0.95), whereas MP3‐Cap (0.70), MP3U (0.75) and S‐H2 (0.75) had lower AUCs, indicating greater classification difficulty.

## 4 Discussion

Accurate determination of skeletal maturation stages is essential in orthodontics, particularly for guiding growth‐modification treatments [15]. The success of orthopaedic interventions, such as functional appliances, face mask therapies and maxillary expansions, largely depends on initiating treatment at the appropriate stage of skeletal development [16, 17, 18, 19]. Identifying pre‐peak and peak growth phases is critical, as orthopaedic corrections are most effective when performed before or during the pubertal growth spurt. Therefore, precise and objective assessment of these maturation stages is crucial for optimising clinical outcomes [20]. To visualise how this approach could be implemented into routine orthodontic practice, Figure S2 presents a schematic diagram outlining the clinical workflow from radiograph acquisition to AI‐based analysis and result delivery.

This study is the first to introduce a deep learning‐driven automated framework for evaluating widely used HWM classifications in orthodontics, incorporating Björk's nine‐stage classification, Hägg and Taranger's five‐stage method, Chapman's AS bone classification, and two modified versions of Björk's system (one incorporating the hook of the hamate bone and the other categorising maturation into pre‐peak, peak and post‐peak phases) to determine which approach offers the highest predictive reliability for identifying key growth phases.

Previous studies aiming to assess skeletal maturation using artificial intelligence on hand, wrist and cervical vertebrae radiographs have employed a wide range of deep learning architectures. These include AlexNet [21], ConvNeXtBase‐296 [22] and AggregateNet [23], which utilise convolutional layers for automated feature extraction. More advanced models have incorporated 3D convolutional backbones [24] and hybrid attention mechanisms such as Attention U‐Net [14] and transformer‐based architectures [12] designed to improve spatial understanding and pixel‐by‐pixel contextual awareness in image analysis. In addition, ensemble deep learning models [11] and various convolutional neural networks (CNNs) [25] remain commonly used due to their strong performance in radiographic image classification.

In this study, YOLOv8 was chosen as the detection framework due to its advanced real‐time detection and segmentation capabilities, which are particularly effective in medical imaging tasks. Its improved feature extraction and ability to handle both classification and segmentation make it well suited for skeletal maturation assessment, where accurate anatomical localization is critical [26]. The effectiveness of YOLOv8 has also been demonstrated in various clinical applications, including paediatric fracture detection, brain tumour localization and identification of endo‐perio lesions in dental radiographs [27, 28, 29]. Building on this architecture, five hand‐wrist maturation methods were evaluated using two variations of the YOLOv8 architecture: a classification‐based model (YOLOv8x‐cls) and a segmentation‐based model (YOLOv8x‐seg). For Groups I and V, the YOLOv8x‐cls model was used to process the entire hand‐wrist radiograph without manual annotation, enabling the network to analyse multiple regions of interest simultaneously through global feature extraction. In contrast, Groups II, III and IV employed a flexible, region‐specific approach using YOLOv8x‐seg, in which predefined anatomical landmarks—such as the MP3 region, adductor sesamoid bone and hamate bone—were manually labelled using polygonal segmentation modules. This allowed the model to focus on specific areas that are critical for the respective skeletal maturation methods.

Björk's [7] HWM method is one of the most widely used techniques in clinical practice for skeletal age assessment, primarily relying on the epiphyseal–diaphyseal relationship of phalanges and radius [30, 31]. This classification system consists of nine stages allowing for a detailed and comprehensive evaluation of skeletal maturation. The evaluation of Group I, based on Björk's HWM method, revealed strong classification performance, particularly in the early and late skeletal maturation stages, where the model achieved the highest predictive reliability. These phases, representing early skeletal development and fully matured stages, were classified with high accuracy and F1 scores exceeding 0.90, highlighting the model's ability to distinguish well‐defined ossification events. However, the classification performance was lower in the stages close to the pubertal growth peak (S‐H2) and at the peak period itself (MP3‐Capping), with F1 scores in the range of 0.72–0.74. This decrease in performance can be attributed to the transitional nature of skeletal maturation during this phase, where morphological changes occur rapidly yet progressively, making stage differentiation more challenging. The overlapping characteristics of adjacent stages, particularly in the transition from pre‐peak to peak growth, may have contributed to increased misclassification rates.

Kim et al. [12] developed an AI‐based automated system for assessing Fishman's 11‐stage SMI [8, 32] and reported an initial overall prediction accuracy of 0.599, which improved to 0.772 after algorithm adjustments. Their findings indicated that prediction accuracy for stage 5 (0.190) was significantly lower compared to other stages, which was attributed to the absence of radiographs classified as SMI 5 in the test dataset. However, even in the primary validation with a larger dataset, SMI 5 still exhibited low accuracy, suggesting that certain SMI stages, particularly those more prone to deviations from the proposed sequence, are inherently associated with higher interobserver variability and reduced prediction reliability. Additionally, the limited number of samples for certain SMI stages was identified as a potential limitation, which may have influenced the measured accuracy of these subgroups.

In another study [11] that utilised Fishman's 11‐stage SMI [8, 27] as the reference standard based on CVM, substantial variability in classification performance was observed across different maturation stages. The precision values for SMI stages 1 to 11 were reported as follows: 86.2%, 23.3%, 11.5%, 53.1%, 9.3%, 21.6%, 50.0%, 28.0%, 18.5%, 41.7% and 85.7%. The lowest precision was found in SMI stages 5 (9.3%) and 6 (21.6%), which correspond to the phases of accelerated growth, suggesting that classification during these stages is more challenging due to limited sample size and an imbalance in the dataset, with a greater number of paediatric patients classified as SMI stage 0.

The evaluation of Group V, which was derived from Björk's [7] HWM method by categorising its nine‐stage classification into three broader phases (pre‐peak, peak and post‐peak periods), demonstrated high classification performance in both the early and late maturation stages. The overall classification accuracy in this group was higher compared to the original nine‐stage Björk system, likely due to the reduced number of classification categories, which minimises misclassification errors between closely related stages. In the original system, subtle skeletal changes between successive stages may lead to overlap and increased classification variability, whereas grouping them into three broader phases provides a more generalised yet stable classification approach. This simplification allows the model to capture major skeletal development trends rather than fine‐stage transitions, resulting in higher predictive reliability and reduced confusion in borderline cases.

The evaluation of Group II, based on Hägg and Taranger's [9] HWM method, demonstrated high classification performance across all maturation stages. Unlike broader classification systems that analyse multiple skeletal regions, this method focuses exclusively on the third finger, allowing for a more streamlined and practical assessment of skeletal maturation. This localised approach likely contributed to the model's high classification accuracy, as fewer regions were analysed, minimising potential classification errors caused by subtle variations across different bones. The clear and progressive ossification sequence of the MP3 region enhances the method's reproducibility and reliability, making it a valuable tool for clinicians seeking a simplified yet effective approach to skeletal age estimation.

Previous studies have emphasised the clinical significance of AS bone calcification in predicting pubertal growth acceleration [10, 33, 34], with Björk et al. [7, 35] reporting that sesamoid ossification occurs about one year before peak growth and does not progress further after this period. Similarly, Grave and Brown [36] identified the AS‐2 stage as the point where growth velocity reaches its peak, highlighting sesamoid maturation as a reliable skeletal growth marker. Building upon these findings, Chapman's classification [10] offers a simplified and practical approach by focusing solely on AS bone calcification, eliminating the variability introduced by multi‐site skeletal assessments. The model demonstrated high classification performance, particularly in later ossification stages where structural changes are more distinct. However, earlier stages showed relatively lower performance, likely due to subtle skeletal changes that make differentiation more challenging. Despite this, the method's simplicity and reproducibility make it a clinically valuable tool for skeletal maturation assessment.

Gonca et al. [4] classified HWR's into three skeletal growth phases—accelerating, high‐velocity and decreasing—based on Fishman's 11‐stage SMI and developed five predictive models incorporating fractal dimension (FD) analysis and additional skeletal and demographic parameters. Their results indicated that the predictive accuracy was relatively low in the first model, which used only FD analysis (0.492–0.656), but increased significantly (0.894–0.927) when Chapman's sesamoid bone classification was added in model 2. This finding highlights the diagnostic significance of the AS bone, demonstrating that despite its small size, its predictable ossification pattern, sharp transitional stages, and clear radiographic visibility make it a highly reliable skeletal maturation indicator.

Previous studies have highlighted the importance of hamate bone calcification in predicting pubertal growth acceleration. Grave and Brown [31] reported that the onset of H1 calcification marks the acceleration phase of the pubertal growth spurt, while the peak growth velocity coincides with the H2 stage. Similarly, Björk [7, 30] identified a strong correlation between the hook of hamate calcification and the pubertal growth peak, emphasising its potential as a reliable skeletal maturation indicator. Lee [37] reported that the initial ossification of the hook of the hamate and pisiform either preceded or coincided with the peak height velocity in the majority of both male and female subjects. Building upon these findings, the hamate bone classification method offers a practical and easily applicable approach, as its distinct anatomical structure allows for clear visual identification of maturation stages. The model demonstrated consistently high classification performance across all maturation stages, with accuracy values ranging from 0.96 to 0.99. The method's simplicity, reproducibility and clear radiographic visibility further enhance its clinical applicability, making it a reliable alternative for skeletal age assessment. Given its strong classification performance and practical advantages, integrating AI‐based automation with hook of hamate bone maturation analysis presents a novel and objective approach, marking an important contribution to the literature in the field of skeletal growth assessment.

Liu et al. [14] developed an AI‐based multi‐task learning framework for automated segmentation and classification of distal radius and ulna (DRU) in HWR's. Similar to the present study, this approach focused on a specific skeletal region, but analysed the distal radius and ulna rather than the hamate or AS bones in our study. Their model achieved high accuracy (94.3% for Radius, 90.8% for Ulna), comparable to the performance observed for the hamate (96%–99%) and AS (95%–97%) bones classifications in the present study. These findings reinforce that AI models achieve greater reliability when applied to distinct anatomical landmarks, minimising classification variability.

One limitation of this study was the reduction in sample size for Group I to maintain an equal number of cases in each stage. This adjustment was necessary because the number of individuals in the peak pubertal growth phase and its preceding stage was lower compared to other stages, which could have led the deep learning model to overfit to more represented stages while underfitting the less frequent ones. By balancing the dataset, the risk of biassed feature extraction and reduced generalisability, particularly in critical growth phases, was minimised. Additionally, the retrospective nature of the study may have introduced selection bias, as the dataset consisted solely of orthodontic patients from a single institution, lacking diversity in ethnic backgrounds and clinical sources, which may limit its representation of the general population. Lastly, although the deep learning models demonstrated high classification performance, variations in image quality and potential radiographic artefacts could still influence prediction accuracy in clinical practice.

## 5 Conclusion

This study demonstrated the strong potential of AI‐driven deep learning models in assessing skeletal maturation using five hand‐wrist classification methods. Among these, Hägg and Taranger's MP3 classification and the calcification stages of the hook of the hamate bone were incorporated into AI‐based analysis for the first time, expanding the scope of automated skeletal maturation assessment and enhancing its applicability in orthodontic diagnostics. The findings demonstrated that AI models achieved high classification performance, particularly in well‐defined anatomical structures such as the MP3 region, AS and hamate bone, which can be easily identified even by general dentists. The ability to accurately assess skeletal maturation in these regions underscores the clinical value of AI in optimising growth phase identification, facilitating timely orthodontic interventions, and improving patient access to appropriate treatments.

## Author Contributions

Conceptualization: S.Ö. Data curation: S.Ö., S.T. Funding acquisition: S.Ö., S.T. Investigation: S.Ö., S.T. Methodology: S.Ö. Project administration: S.Ö. Resources: S.Ö., S.T. Supervision: S.Ö. Validation: S.Ö. Visualisation: S.Ö., S.T. Writing – original draft: S.Ö., S.T. Writing – review and editing: S.Ö.

## Ethics Statement

This retrospective study received ethical approval from the İnönü University Non‐Interventional Clinical Research Ethics Committee (Approval No: 2023/5340, Date: 26.12.2023).

## Consent

Informed consent forms were obtained from all patients and, in the case of participants under the age of 16, from their parents or legal guardians prior to the initiation of treatment; however, no additional consent was required due to the retrospective nature of the study.

## Conflicts of Interest

The authors declare no conflicts of interest.

## Supporting information


**Figure S1:** Combined ROC curves for the five skeletal maturation assessment methods used in this study. The area under the curve (AUC) values indicate the overall predictive accuracy of each method, with higher AUC values reflecting better classification performance.
**Figure S2:** Schematic representation of the clinical workflow for AI‐assisted skeletal maturation assessment, showing the process from hand‐wrist radiograph acquisition to automated analysis and result delivery to healthcare providers.

## Data Availability

The data that support the findings of this study are available from the corresponding author upon reasonable request.
