# Supplementary material for: Deep Learning Based Evaluation of Skeletal Maturation: A Comparative Analysis of Five Hand‐Wrist Methods
Source: Orthod Craniofac Res. 2025 Jul 24;28(6):943–54. doi: 10.1111/ocr.70008 (PMC12603682; doi:10.1111/ocr.70008)
Supplement: Supplementary file 1 — Figure S1: Combined ROC curves for the five skeletal maturation assessment methods used in this study. The area under the curve (AUC) values indicate the overall predictive accuracy of each method, with higher AUC values reflecting better classification performance. Figure S2: Schematic representation of the clinical workflow for AI‐assisted skeletal maturation assessment, showing the process from hand‐wrist radiograph acquisition to automated analysis and result delivery to healthcare providers. [file OCR-28-943-s001.docx]

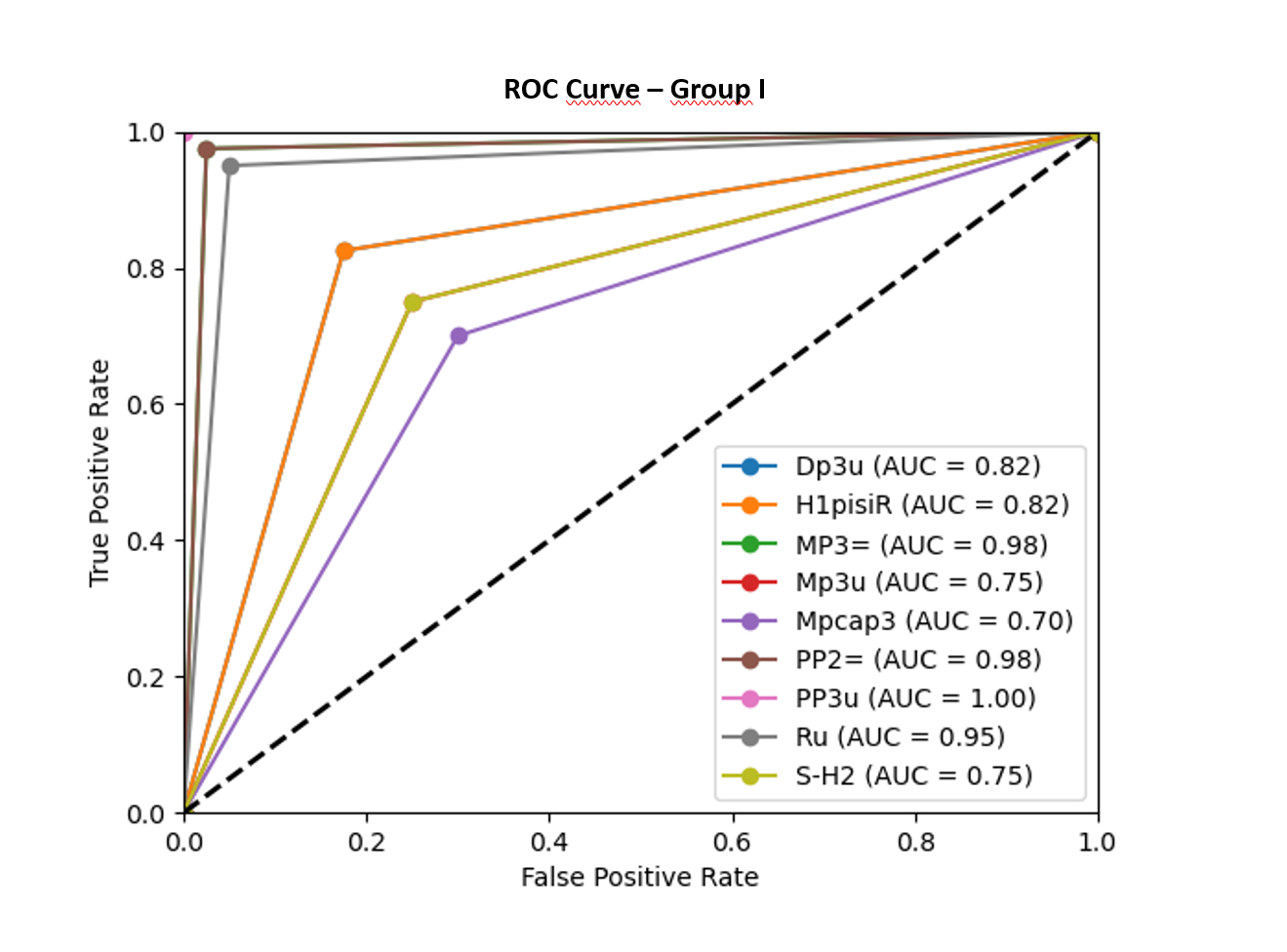

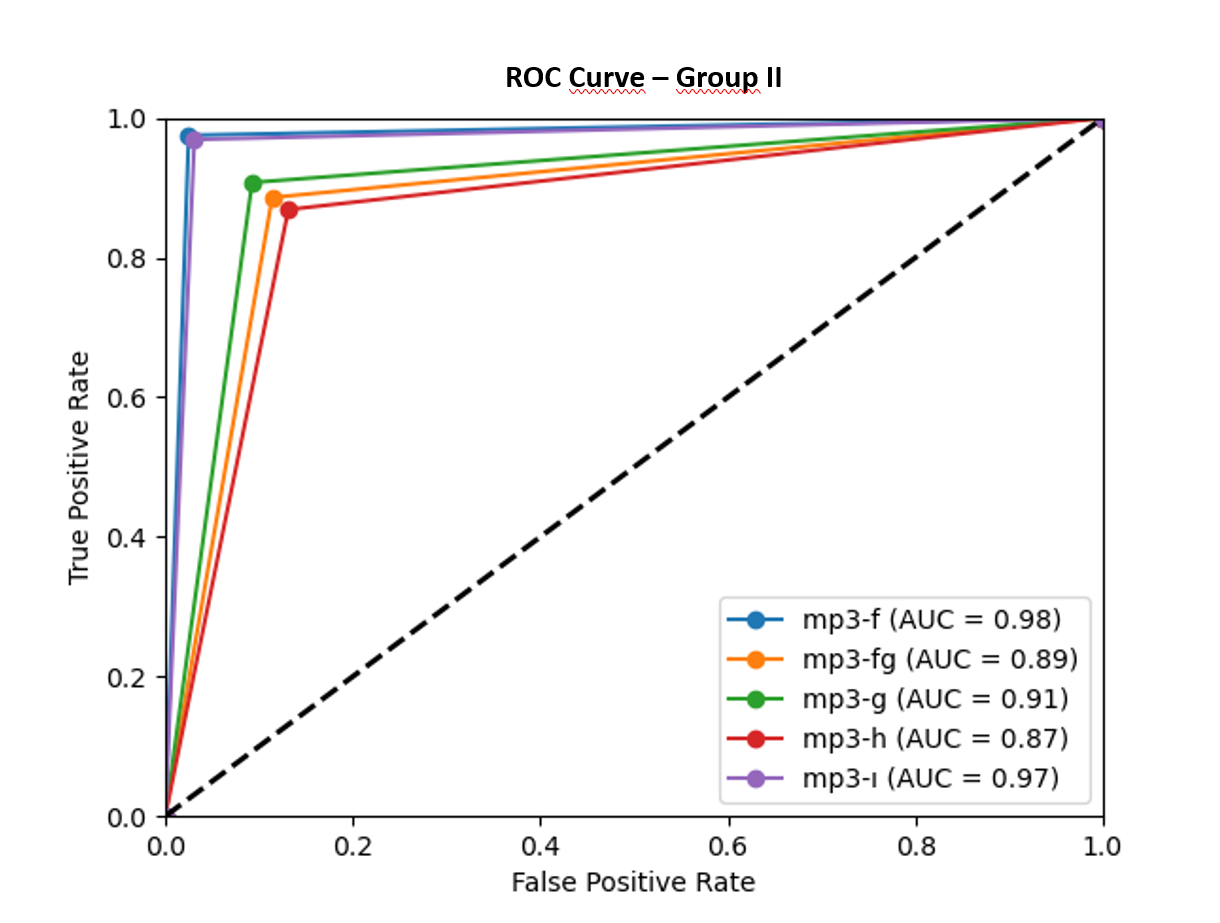

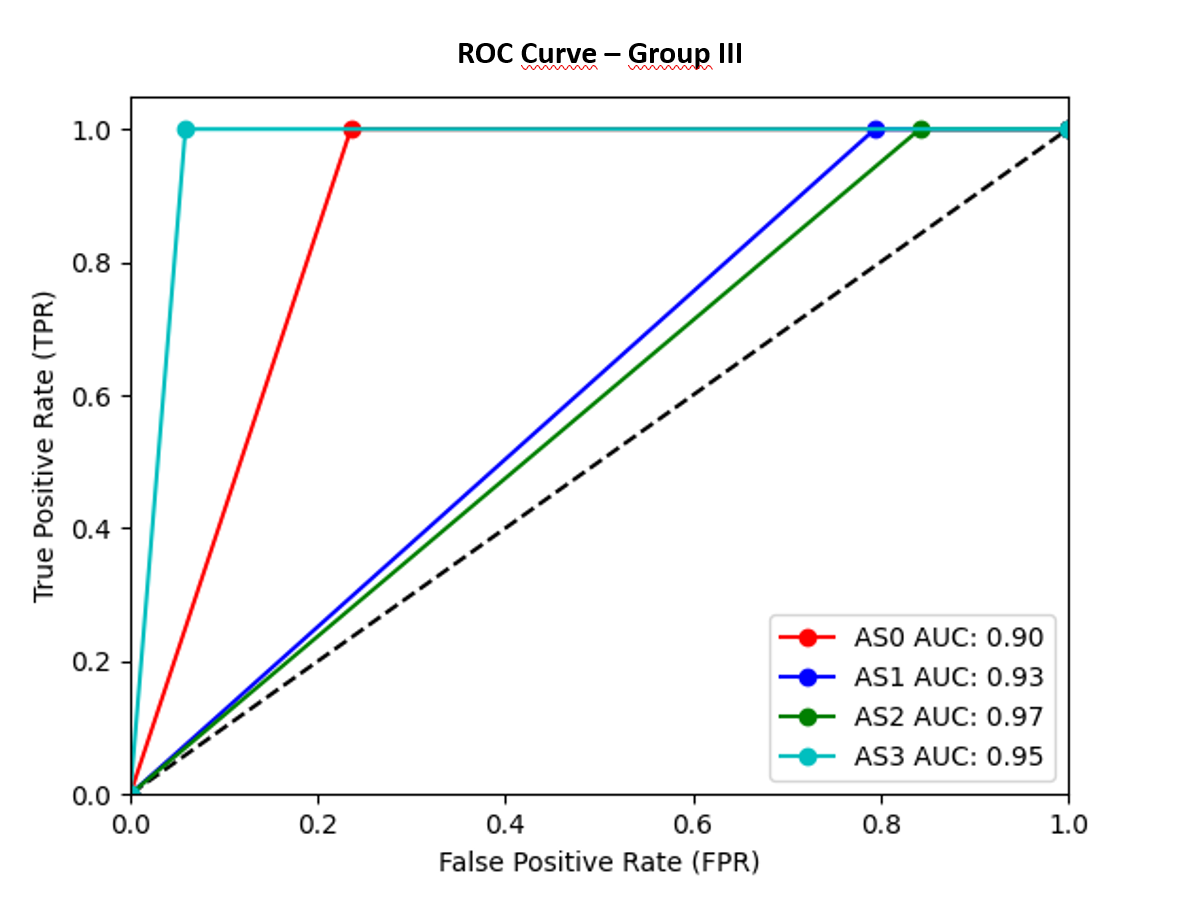

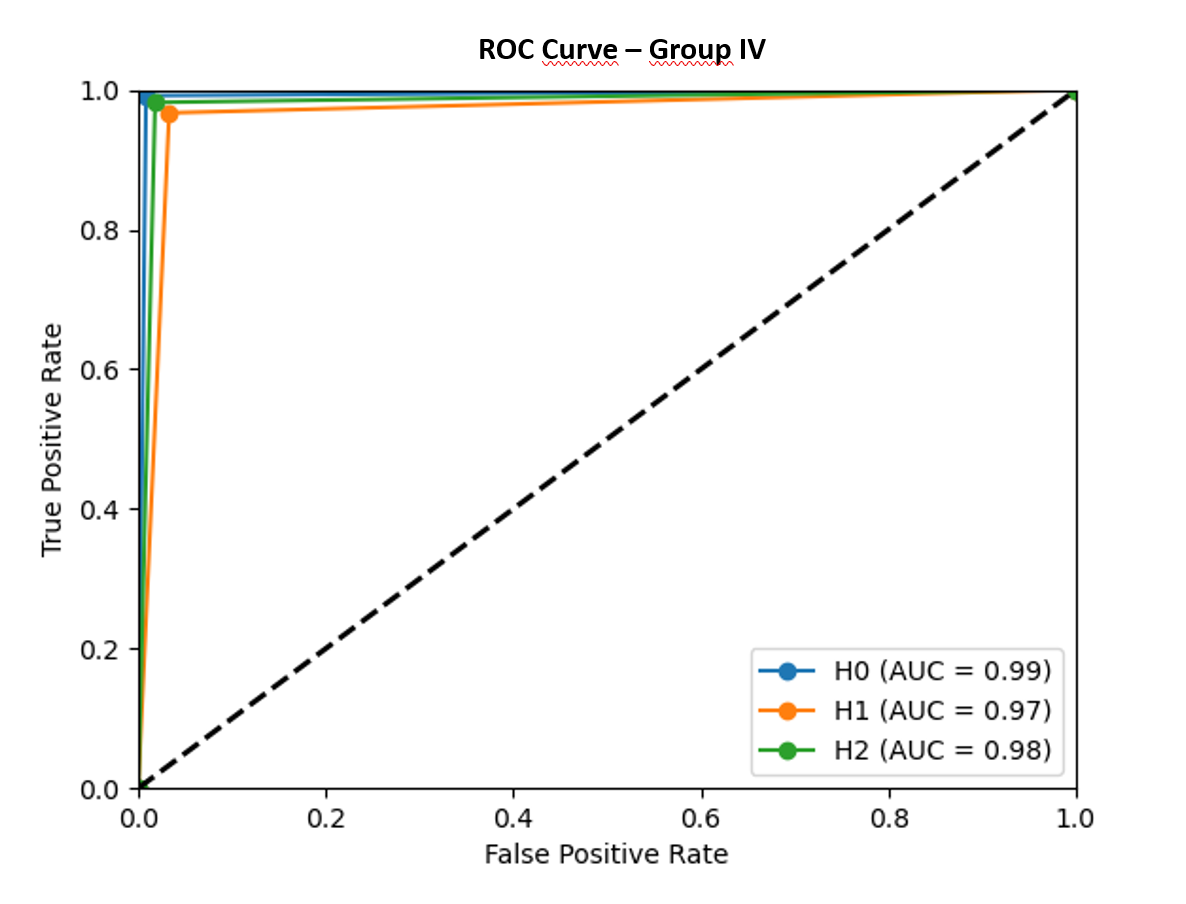

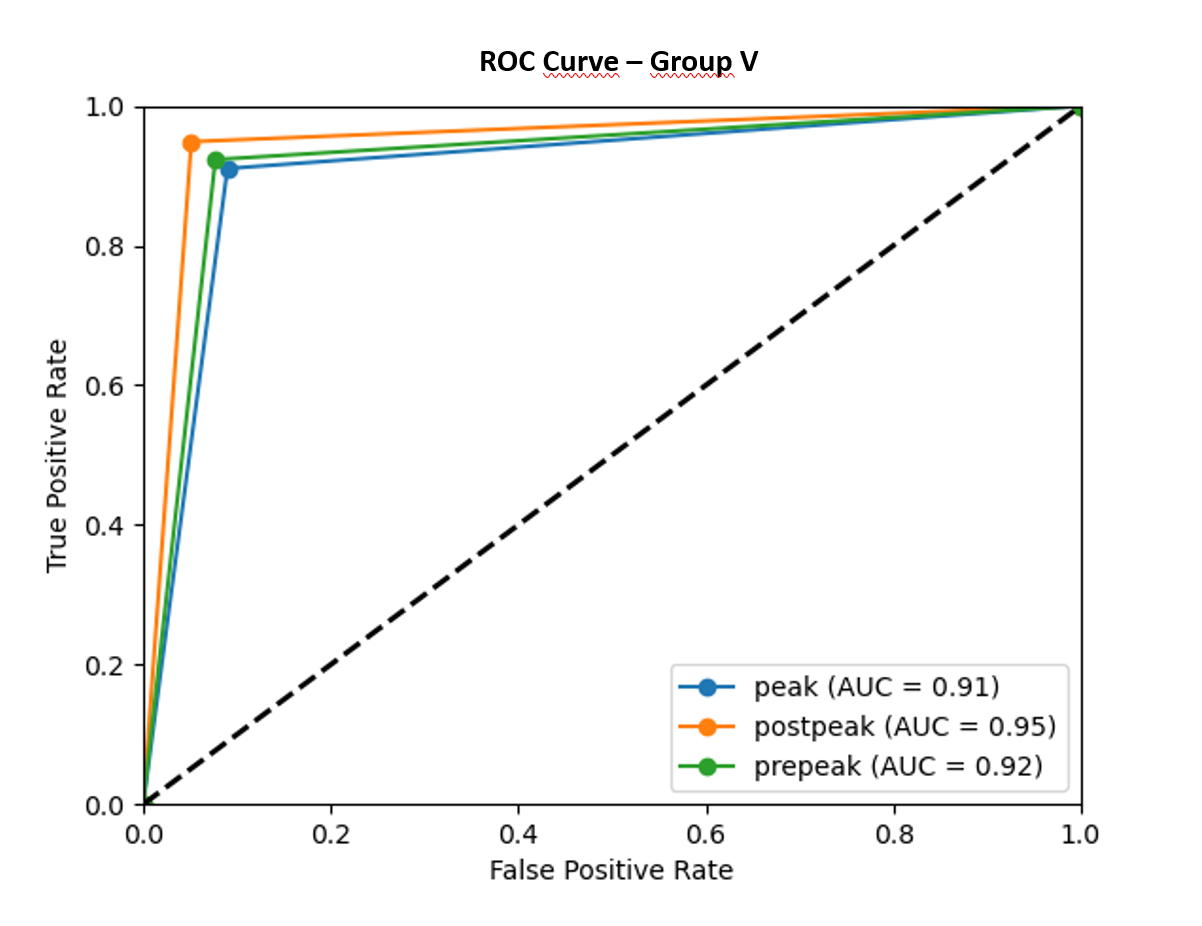


**Figure S1:** Combined ROC curves for the five skeletal maturation assessment methods used in this study. The area under the curve (AUC) values indicate the overall predictive accuracy of each method, with higher AUC values reflecting better classification performance.


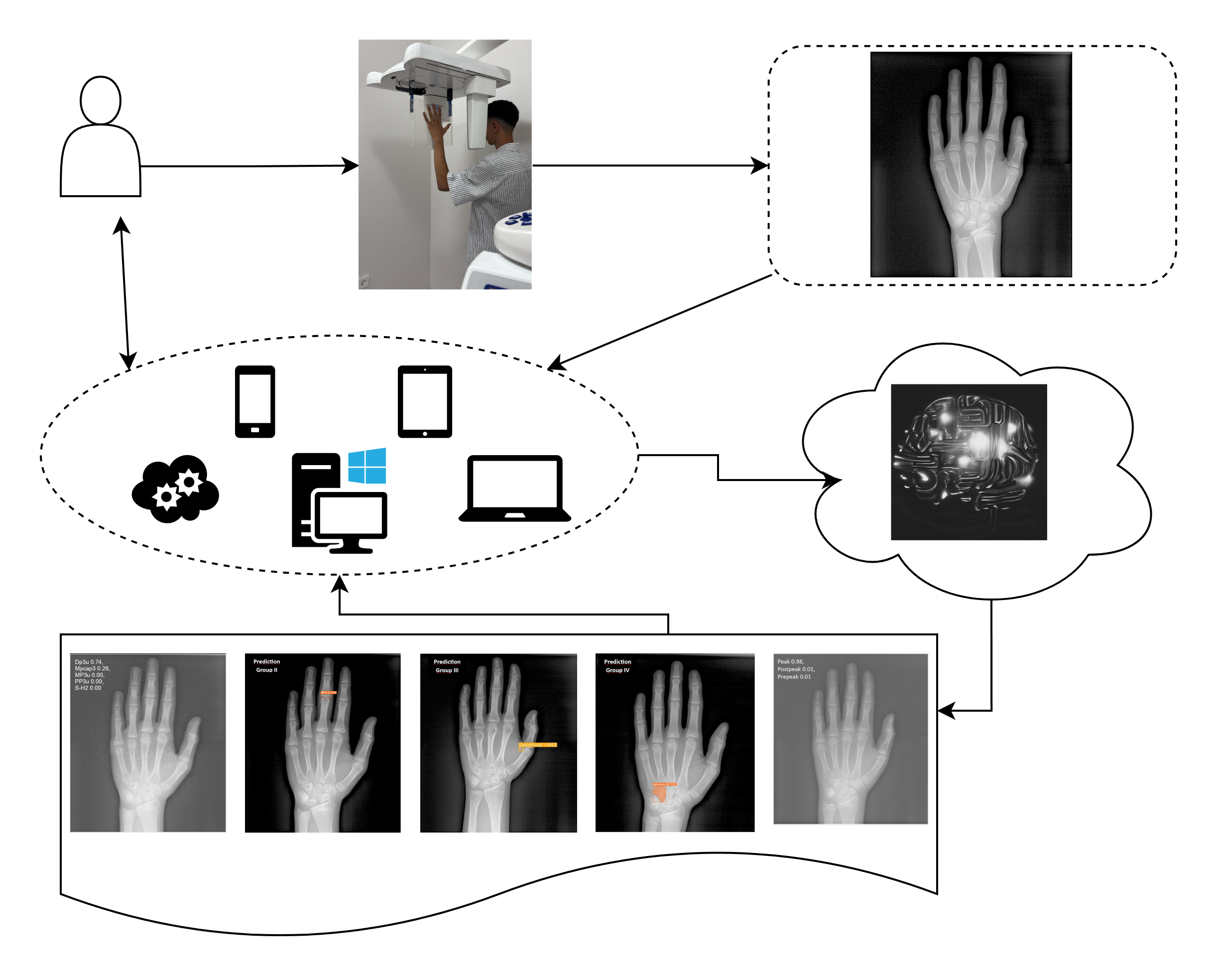


**Figure S2:** Schematic representation of the clinical workflow for AI-assisted skeletal maturation assessment, showing the process from hand-wrist radiograph acquisition to automated analysis and result delivery to healthcare providers.
